# *EHP*’s New Manuscript Submission System

**DOI:** 10.1289/ehp.11926

**Published:** 2008-08

**Authors:** Hugh A. Tilson

**Affiliations:** *EHP*, E-mail: tilsonha@niehs.nih.gov

In the May 2008 issue of *EHP*, I indicated in the editorial “Recent Changes at *Environmental Health Perspectives*” that we were planning to implement a new web-based manuscript submission and tracking system. I am pleased to announce that, as of 15 July 2008, all manuscripts submitted to the journal are being handled by ScholarOne’s Manuscript Central. We are joining more than 170 societies and publishers (> 1,850 books and journals) in using this system.

At the present time, all commentaries, reviews, and research articles are initially screened by an advisory board to determine if they meet criteria relative to originality, scientific quality, environmental significance, appropriate degree of speculation, clarity of presentation, and conciseness. About 60% of the 1,200 manuscripts submitted to the journal each year are rejected without further review. Those papers selected for peer review are assigned to an associate editor, who is responsible for identifying reviewers and coordinating the peer review of the manuscript. Ultimately, the Associate Editors recommend to the editor-in-chief whether a paper should be published in *EHP*. Approximately 40% of all peer-reviewed papers are accepted for publication.

In 2007, we determined that the average time to first decision for papers was approximately 2 months, and the average time from submission to acceptance was about 6.5 months. Our goal is to shorten the time between submission and acceptance of manuscripts by 20%. We believe that Manuscript Central will help us achieve that goal by streamlining what is currently a labor-intensive process. It will also significantly improve our ability to track manuscripts during the review process and provide critical information to the editor-in-chief and associate editors concerning expertise and performance of potential reviewers.

Before submitting a paper for the first time using the new system, authors will need to register with Manuscript Central at http://mc.manuscriptcentral.com/ehp, where they will be asked to provide five or more keywords concerning their research expertise and interests. Then authors will be guided through a few simple steps to submit their papers.

Once a paper is in the system, it will be tracked automatically by Manuscript Central. Because this system is web-based, all associate editors will be able to access the database to determine the status of papers assigned to them and retrieve information concerning potential reviewers. Of course, Manuscript Central is password protected, and information concerning authors and reviewers will be available only to the editor-in-chief and the relevant associate editors.

We at *EHP* are excited by the opportunities afforded by the implementation of Manuscript Central. We look forward to continuing to receive high-quality research papers on environmental health sciences and the satisfaction of knowing that we are publishing the best research in the most timely and efficient manner possible.

## Figures and Tables

**Figure:**
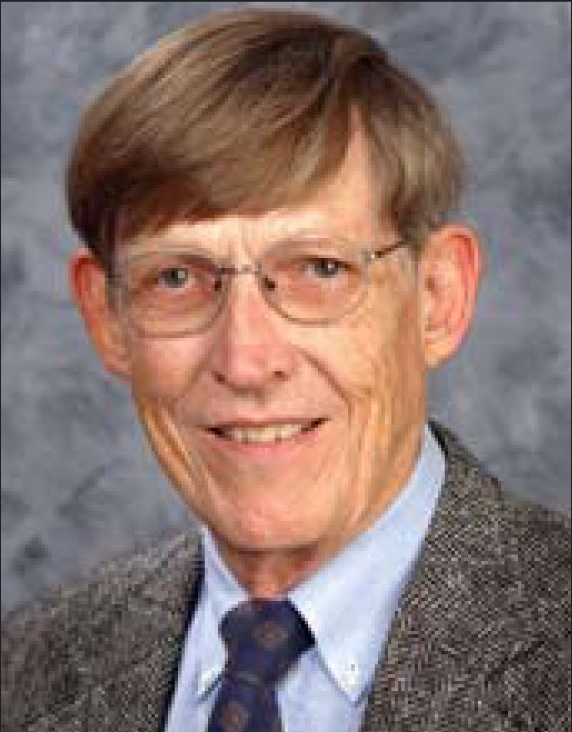
Hugh A. Tilson

